# Genetic dissection of endothelial transcriptional activity of zebrafish aryl hydrocarbon receptors (AHRs)

**DOI:** 10.1371/journal.pone.0183433

**Published:** 2017-08-17

**Authors:** Wade W. Sugden, Roberto C. Leonardo-Mendonça, Darío Acuña-Castroviejo, Arndt F. Siekmann

**Affiliations:** 1 Max Planck Institute for Molecular Biomedicine, Muenster, Germany; 2 Cells-in-Motion Cluster of Excellence (EXC 1003 –CiM), University of Muenster, Muenster, Germany; 3 Universidade Atlântica, Fábrica da Pólvora de Barcarena, Barcarena, Portugal; 4 Instituto de Biotecnología, Centro de Investigación Biomédica, Parque Tecnológico de Ciencias de la Salud, Universidad de Granada, Granada, Spain; 5 Unidad de Gestión Clínica de Laboratorios Clínicos, CIBER de Fragilidad y Envejecimiento, Ibs.Granada, Complejo Hospitalario de Granada, Granada, Spain; Texas A&M University, UNITED STATES

## Abstract

The aryl hydrocarbon receptor (AHR) is a basic helix-loop-helix transcription factor conserved across phyla from flies to humans. Activated by a number of endogenous ligands and environmental toxins, studies on AHR function and gene regulation have largely focused on a toxicological perspective relating to aromatic hydrocarbons generated by human activities and the often-deleterious effects of exposure on vertebrates mediated by AHR activation. A growing body of work has highlighted the importance of AHR in physiologic processes, including immune cell differentiation and vascular patterning. Here we dissect the contribution of the 3 zebrafish AHRs, *ahr1a*, *ahr1b* and *ahr2*, to endothelial *cyp1a1/b1* gene regulation under physiologic conditions and upon exposure to the AHR ligand Beta-naphthoflavone. We show that in fish multiple AHRs are functional in the vasculature, with vessel-specific differences in the ability of *ahr1b* to compensate for the loss of *ahr2* to maintain AHR signaling. We further provide evidence that AHR can regulate the expression of the chemokine receptor *cxcr4a* in endothelial cells, a regulatory mechanism that may provide insight into AHR function in the endothelium.

## Introduction

The aryl hydrocarbon receptor (AHR) was originally identified for its role in mediating toxicity of environmental compounds [1]. Dubbed the “dioxin receptor,” AHR is a ligand-activated transcription factor that binds a number of halogenated aromatic hydrocarbons (HAHs) and polycyclic aromatic hydrocarbons (PAHs) [2]. Many of these ligands are environmental toxins like dioxin (see below), but others are derived from compounds in food, gut microbiota, and circulating lipids/hormones. In its unliganded form, AHR is confined to the cytoplasm in a complex with two HSP90 proteins, a p23 and X-associated protein (XAP) chaperones [3]. When ligand bound, conformational changes allow AHR to dissociate from the cytoplasmic complex and bind to a cofactor, aryl hydrocarbon nuclear translocator (ARNT, also called HIF-1B), and together these proteins translocate to the nucleus and bind AHR-response elements in the promoters of target genes [4, 5]. While the AHR has many transcriptional targets, the two best characterized are the cytochrome P450 1A1 and 1B1 (CYP1A1 and CYP1B1) genes [6]. These enzymes belong to the CYP1 family of monoxygenases, and are responsible for the metabolism of a variety of endogenous and xenobiotic molecules into bioactive compounds and toxic derivatives, respectively.

The “dioxin receptor” was so-called because of its role in mediating the carcinogenic effects of the environmental contaminant 2,3,7,8-Tetrachlorodibenzo-*p*-dioxin (TCDD) [7]. The affinity of AHR for TCDD is extremely high, and the dissociation constant (K_d_) for binding is in the order of picomolar concentrations of TCDD [8]. In comparison, the PAH Beta-naphthoflavone (BNF) is about 100x less potent, and the tryptophan metabolite kynurenine (an AHR agonist *in vivo*) binds AHR with a K_d_ of ~4 uM [9, 10]. The Drosophila homologue cannot bind dioxin [11], which -combined with the fact that common environmental toxins were not found before widespread human development- suggests that AHR has physiological roles besides regulating xenobiotic metabolism.

AHR-deficient mice indicate physiological functions of AHR signaling within the vasculature. Notably, AHR -/- mice have smaller livers, displaying portosystemic shunting that prevents blood from circulating through the organ [12]. This phenotype is the result of an intact ductus venosus, a fetal vascular connection between the portal vein and inferior vena cava that must be closed at birth. This event requires AHR function in ECs, as EC-specific Tie2:Cre-mediated deletion reproduces the shunt phenotype, while Albumin:Cre-mdiated deletion in hepatocytes does not [13]. AHR -/- mice also have vascular patterning defects in the eye (ectopic loops) and kidney (decreased capillary density), but these are relatively uncharacterized [12].

Interestingly, shear stress plays a role in the AHR/CYP1A/1B signaling axis. The expression of AHR and cyp1a1/1b1 are positively regulated by shear stress, and nuclear translocation of AHR is enhanced in areas of high shear [14, 15]. Han et al. showed that CYP induction by flow required AHR binding to its promoter, and that flow-induced cell cycle inhibition required AHR. CYP enzymes can metabolize lipids like arachnidonic acid into vasoactive HETEs and EETs that modulate vessel tone [16]. Accordingly, AHR -/- and CYP1B1 -/- mice have low blood pressure, while CYP1A1 -/- animals are hypertensive [17, 18]. Kynurenine, a known endogenous AHR ligand, exhibits vasodilatory properties [19]. Still, the role of AHR in developmental vascular patterning is unknown.

The zebrafish model has been used primarily to investigate AHR-mediated toxicity of HAHs and PAHs during development. In contrast to mammals, which contain a single AHR gene, the zebrafish genome has 3 AHR co-orthologues. Phylogenetic analysis supports a model where a single ancestral AHR gene in invertebrates underwent a duplication to yield an AHR1 and AHRR, and these are present in all chordate lineages. A subsequent duplication of AHR1 led to two AHR genes (AHR1/AHR2) [20]. Mammals are thought to have lost the AHR2 gene. Fish retained the AHR1/AHR2 gene pair, and this was expanded by the whole genome duplication that occurred in the lineage of ray-finned fish after the separation of bony and cartilaginous fish [21], yielding 4 co-orthologues: AHR1a/AHR2a and AHR1b/AHR2b. In the zebrafish *Danio rerio*, one of these co-orthologues has been lost, yielding 3 AHR genes that have been identified: *ahr1a*, *ahr1b* and *ahr2* [22]. TCDD administration causes pericardial edema, growth failure, circulation loss, craniofacial malformations and death in zebrafish embryos [23]. AHR -/- mice are protected from these deleterious effects of dioxin [24]. Of the zebrafish AHRs, *ahr2* is responsible for most TCDD-induced toxicity and knocking it down dramatically improves survival [25]. However, data on vascular patterning in *ahr2-*deficient zebrafish sans exposure to toxic chemicals is scarce. A flow defect has been reported in a subset of cranial vessels in embryos exposed to AHR agonists, along with some subtle patterning variations [26–28]. Recently, it has been shown that *ahr1b* can functionally compensate for the loss of *ahr2* in the endothelium to upregulate *cyp1a1* in response to exogenous AHR ligands [29]. However, no analysis was done in these embryos without exposure to chemicals, and there is no report of vascular patterning defects in zebrafish lacking all AHR function.

## Materials and methods

### Fish lines

Zebrafish were housed in an aquaculture facility maintained at 28.5°C with a 14 hrs day/night cycle, as per standard fish husbandry practice [30]. Fish carrying the vascular transgene *Tg(kdrl*:*EGFP)*^*s843*^ [31] were used to examine vessel morphologies in live embryos. *ahr2*^*hu3335*^ mutants used in this study were described previously [29]. All animal experiments were performed in compliance with the relevant laws and institutional guidelines of the Max Planck Institute for Molecular Biomedicine and were approved by local animal ethics committees of the Landesamt für Natur, Umwelt und Verbraucherschutz Nordrhein-Westfalen.

### Live imaging and drug treatments

Embryos were maintained at 28.5°C in E3 medium, supplemented with 0.003% phenylthiourea to prevent pigment formation. For live imaging experiments, embryos were anaesthetized in 1x tricaine (168 mg/L). Imaging was done on an inverted Sp5 (Leica Microsystems) confocal microscope, with embryos mounted in 1% low melting point agarose (Life Technologies) in glass bottom dishes (Wilco Wells). Embryos from ISH experiments were stored in 75% glycerol/PBST and imaged on standard stereo dissection microscopes, with high magnification images taken on a Zeiss AxioImager microscope. For drug treatments, nifedipine (Sigma-Aldrich) and Beta-naphthoflavone (Sigma-Aldrich) were dissolved in DMSO to 10 mM stocks, and diluted in E3 medium to experimental concentrations listed. For nifedipine treatments, 2x tricaine was also included to anaesthetize embryos. Treatments were administered to embryos in 24-well plates, with 25 embryos/well in 1mL of E3.

### TALEN mutagenesis

All of the mutants generated in the course of this study were made by TALEN mutagenesis. TALEN pairs were designed using the online TAL Effector Nucleotide Targeter (https://tale-nt.cac.cornell.edu/). Criteria for optimal TALEN pairs were followed as described in Cermak et al., 2011 [32]. The following TALEN pairs were selected for optimal length and presence of a restriction site in the spacer.

*ahr1a* 5’ TALEN arm

DNA sequence: 5’-gaagttctggccagcttggc-3’

RVD sequence: NH NI NI NH NG NG HD NG NH NH HD HD NI NH HD NG NG NH NH HD

*ahr1a* 3’ TALEN arm

DNA sequence: 5’-tccacaggagatcgtatcc-3’

RVD sequence: NH NH NI NG NI HD NH NI NG HD NG HD HD NG NH NG NH NH NI

*ahr1b* 5’ TALEN arm

DNA sequence: 5’-cggttaaactctgag-3’

RVD sequence: HD NN NN NG NG NI NI NI HD NG HD NG NN NI NN

*ahr1b* 3’ TALEN arm

DNA sequence: 5’-tcttcttccatttcc-3’

RVD sequence: NN NN NI NI NI NG NN NN NI NI NN NI NI NN NI

The Golden Gate TALEN kit v2.0 used for generation of TALENs was a gift from Daniel Voytas and Adam Bogdanove (Addgene kit # 1000000024). *ahr1a* and *ahr1b* TALENs were assembled using NH RVD module plasmids instead of NN RVDs, and cloned directly into the GoldyTALEN vector [33]. TALENs were linearized with SacI and mRNA produced with T3 mMessage Machine kit (Ambion). mRNA for each TALEN arm was co-injected (1:1 mix of 5’:3’ TALEN arm) into 1-cell stage WT embryos. TALEN efficacy was checked by DNA extraction from 20 pooled embryos from each injection (see **Genotyping** section for details). PCR was performed with standard TAQ polymerases with no proof-reading ability, and undigested bands were gel extracted for subsequent TOPO^®^ cloning by TA overhangs and sequencing to identify founder fish and establish stable lines. Total TALEN mRNA injected was 50 pg (*ahr1a*) and 20 pg (*ahr1b*), and ~100 fish raised for the founder (F_0_) generation.

### Genotyping

Standard PCR genotyping protocol was followed with HotMaster TAQ polymerase (PeqLab, 01–8210) using the following primers and conditions:

*ahr1a*: forward primer GGGCGATGTCTAAATCTATTCT and reverse primer GAGAGATTAATGCATAACCAATTT, digested with HinfI. *ahr1b*: forward primer TGTATATTTTCATGCCATCC and reverse primer GTGTTTCGTCTGACCCAG, digested with MspI, *ahr2*: forward primer GCTCAATGTCCCTGGGAACACCTGG and reverse primer CTGAATTCCTGGTTAGCAGCCAAGTTATTCTG.

The annealing temperature for all primer pairs was 60°C. PCR for *ahr1a* yields a 499 bp product, cleaving to 327 bp and 172 bp fragments in WT. PCR for *ahr1b* yields a 447 bp product, cleaving to 255 bp and 192 bp fragments in WT. PCR amplicons for *ahr2* were sequenced to identify the WT and *hu3335* allele.

### *In situ* hybridization

*In situ* hybridization to detect transcripts in whole embryos was performed as previously described [34]. The *cxcr4a* probe was previously described [35]. To generate all other probes, RNA was extracted from pooled 48 hpf WT embryos using the Qiagen Micro RNeasy kit (Qiagen, 74004). cDNA was produced with the iScript cDNA Synthesis Kit from Bio-Rad (Bio-Rad, 1708890), and the following primers were used to amplify gene-specific cDNA using standard TAQ polymerase. *ahr1a*: forward primer CGGCATGAGTTTCAGAGACA and reverse primer AAGAGGCAGGATCAGAAGAT, *ahr1b*: forward primer CCAGAAAGGAGCAGGTACGGATGAAGTTAC and reverse primer GTTGACGGCTGTCTGCGAGAGGG, *ahr2*: forward primer GATGGAGTCAACTTCTCAGAAGGGGAGC and reverse primer ACTACTAGTATCCATTCCCTCTTGGATGTTCATTC, *cyp1a1*: forward primer CGGAAACAACCCACATTTGAGTCTGAC and reverse primer CGGTGAACTTTAACCTTTGCAGCAGGAT, *cyp1b1*: forward primer CGAATGGCTCAGAAATACGGCGAC and reverse primer TGGACCAGCACAGACGTGAAGAGGAA, *glut1*: forward primer GCAGGAGGAACTCAATGCTC and reverse primer TGGACCAGCACAGACGTGAAGAGGAA. These PCR amplicons were TOPO cloned into the pCRII-TOPO TA vector from Invitrogen (Invitrogen, 450640), and digested/sequenced to determine orientation for antisense probe generation following linearization of the plasmid.

### Quantification and statistical analysis

All experiments were performed 2–3 times, with the exception of confocal imaging of the vasculature in live embryos. This was performed once to document the lack of gross vascular defects in *ahr1a -/-* and triple AHR mutants. To analyze different AHR mutants, embryos for ISH experiments were obtained from incrosses of *ahr2*^*hu3335*^ *+/-* or *ahr1a*^*mu153*^ +/-; *ahr1b*^*mu145*^ +/-; *ahr2*^*hu3335*^ +/- adult fish. Embryos from WT incrosses were used for experiments only comparing DMSO and BNF treatments. For analysis of ISH experiments, all embyros were imaged and genotyped. Numbers are reported as “Number of embyros of a particular genotype with staining pattern as in image/total number of embryos of that genotype analyzed”. Statisical analysis for counts of *cxcr4a*-positive cells in the hindbrain was performed using GraphPad Prism 6.0. For live imaging experiments, fish were imaged blindly and genotyped aferwards. All data available from the authors upon request.

## Results

### Zebrafish AHR genes are differentially expressed during development

**Fig 1:**
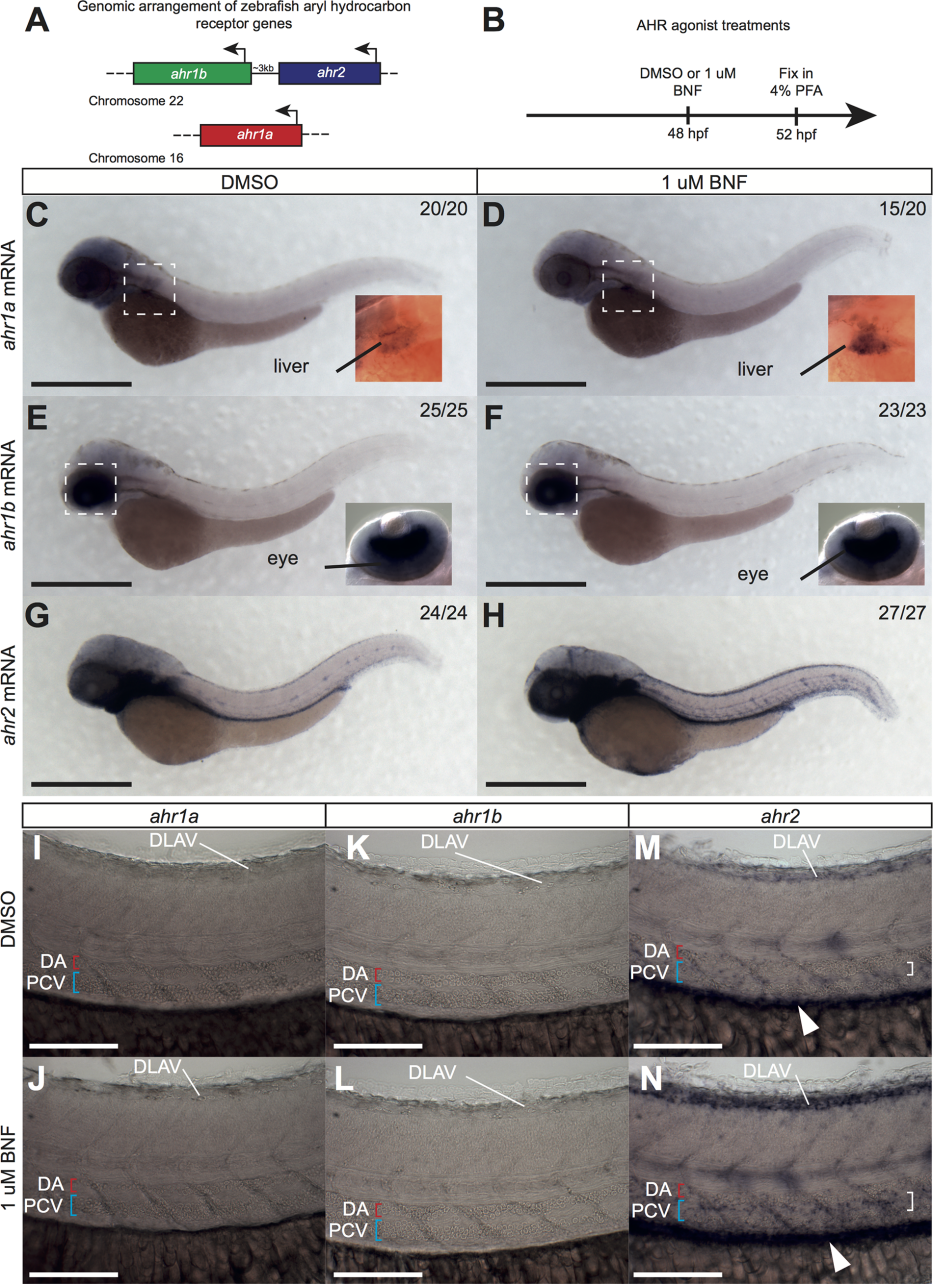
Zebrafish AHR genes are differentially expressed during development. **(A)** Schematic of genomic arrangement of zebrafish AHR genes. Note linkage of *ahr1b* and *ahr2* (~3 kb intergenic distance). (**B)** Experimental design. Embryos were exposed to DMSO or 1 uM BNF for 4 hrs starting at 48 hpf, then fixed in 4% PFA for ISH. (**C-H)** Stereomicroscope images of whole mount ISH in 52 hpf embryos exposed to DMSO or 1 uM BNF showing expression of *ahr1a* (**C, D**), *ahr1b* (**E, F**) and *ahr2* (**G, H**). Boxes indicate region of high magnification image in inset. Numbers indicate embryos with indicated expression pattern/total embryos analyzed. Scale bar is 500 um. (**I-N)** High magnification images of trunk in 52 hpf embryos exposed to DMSO or 1 uM BNF showing expression of *ahr1a* (**I, J**), *ahr1b* (**K, L**) and *ahr2* (**M, N**). No staining evident for *ahr1a* or *ahr1b* in either condition. *ahr2* expression is detected in DLAV, gut (white arrowhead) and region between DA and PCV (white bracket), and is increased by BNF-treatment. Scale bar is 100 um. Abbreviations—AHR: aryl hydrocarbon receptor, BNF: beta-naphthoflavone, DA: dorsal aorta, DLAV: dorsal longitudinal anastomotic vessel, DMSO: dimethylsulfoxide, hpf: hours post fertilization, ISH: *in situ* hybridization, kb: kilobase pair PCV: posterior cardinal vein, PFA: paraformaldehyde.

Whereas mammals possess a single AHR gene, the zebrafish *Danio rerio* contains 3 distinct AHR genes in its genome [22]: linkage of *ahr1b* and *ahr2* on chromosome 22, and a single *ahr1a* on chromosome 16 (**Fig 1A**). To address the role of AHR in embryonic vascular development, we first determined the expression patterns of all 3 AHR genes at 52 hpf by *in situ* hybridization. In addition to endogenous transcripts, we also investigated AHR expression in embryos exposed to the AHR agonist Beta-naphthoflavone (BNF) (**Fig 1B**). We found differential expression of AHR genes in the embryo, with partially overlapping domains and responsiveness to BNF treatment. *ahr1a* mRNA was only weakly detected in the liver, and was upregulated by BNF (**Fig 1C and 1D**). In contrast, *ahr1b* was highly expressed only in the developing eye. This expression was not influenced by BNF (**Fig 1E and 1F**). Finally, *ahr2* was the most broadly expressed of the 3 genes and was strongly upregulated by BNF (**Fig 1G and 1H**). Higher magnification images of the trunk indicate expression of *ahr2*, but not *ahr1a* or *ahr1b*, in the tissue between the dorsal aorta (DA) and posterior cardinal vein (PCV) and around the dorsal longitudinal anastomotic vessel (DLAV) (**Fig 1I–1N**). These results show that different AHR genes are expressed in distinct tissues and respond differently to agonist treatment.

### Differential vascular expression of cytochrome p450 1 genes under basal and BNF-induced conditions

**Fig 2:**
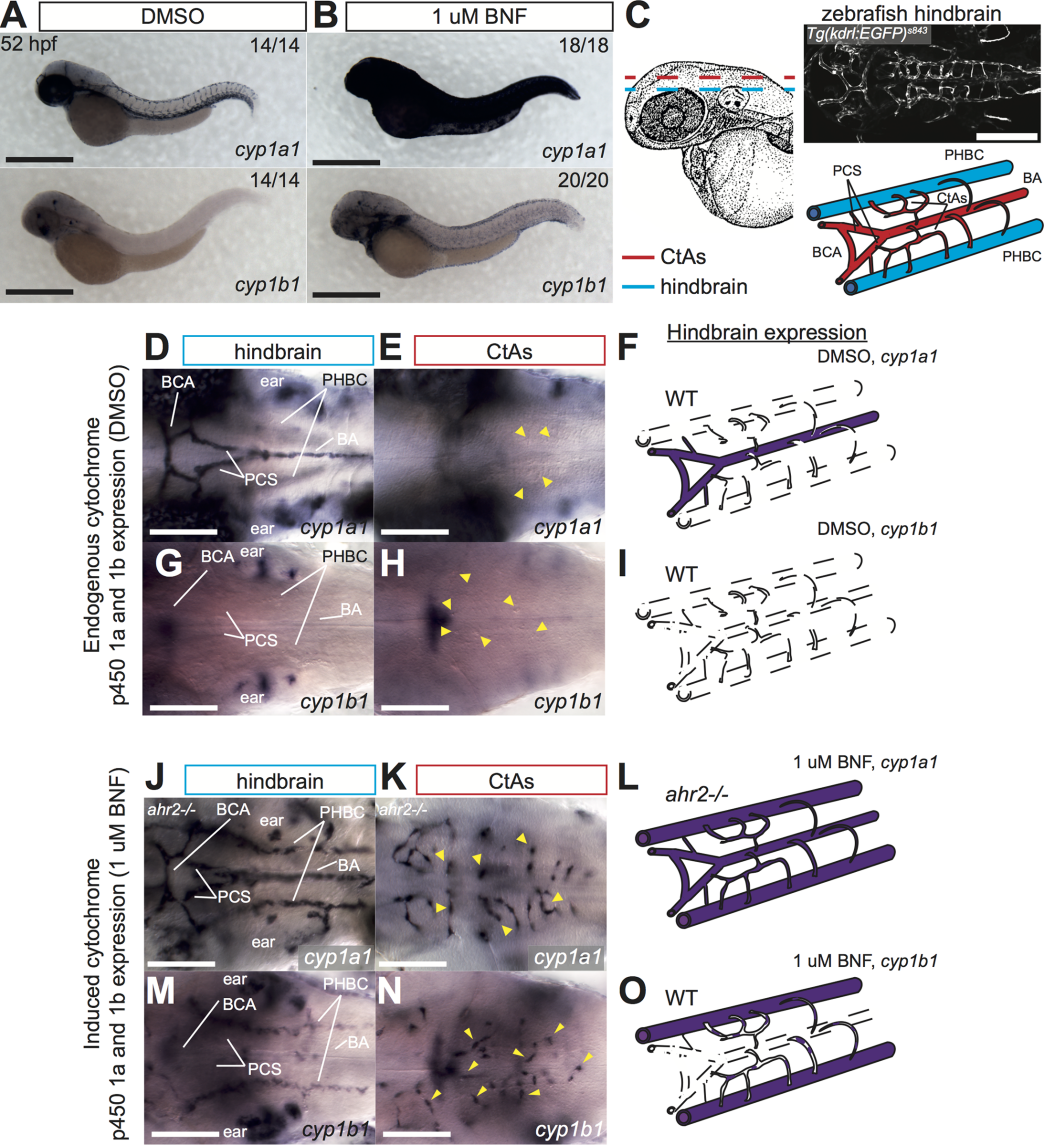
Differential vascular expression of CYP genes under basal and BNF-induced conditions. **(A)** Stereomicroscope images of whole mount ISH for *cyp1a1* and *cyp1b1* in 52 hpf WT embryos after 4 hrs exposure to DMSO (**A**) or 1 uM BNF (**B**). Strong upregulation of *cyp1a1* in skin can be detected. *cyp1b1* is also induced in the skin, but to a lesser extent. Numbers indicate embryos with indicated expression pattern/total embryos analyzed. Scale bar is 500 um. (**C)**. *Camera lucida* drawing of 48 hpf zebrafish embryo from Kimmel, et al 1995 [36]. Dashed lines indicate plane of images to visualize central arteries (CtAs, red) and hindbrain vessels (blue). Confocal image shows dorsal view of hindbrain vasculature of live embryo at 48 hpf (scale bar is 200 um). Cartoon schematic depicts arrangement of hindbrain vessels. **(D-I)** High magnification images of basal *cyp1a1/b1* expression at 52 hpf in the hindbrain (**D, G**) and CtAs (**E, H**) of the head vasculature in embryos treated with DMSO. Schematics in **F,I** summarize hindbrain expression patterns of both genes. (**J-O)** High magnification images of BNF-induced *cyp1a1/b1* expression at 52 hpf in the hindbrain (**J, M**) and CtAs (**K, N**) of the head vasculature in embryos treated with 1 uM BNF. Schematics in **L,O** summarize hindbrain expression patterns of both genes. Analysis of *cyp1a1* expression was performed in *ahr2* mutant embryos to permit visualization of hindbrain vasculature. Yellow arrowheads mark CtAs. Scale bar is 100 um in all high magnification images. Abbreviations–AHR: aryl hydrocarbon receptor, BA: basilar artery, BCA: basal communicating artery, BNF: beta-naphthoflavone, CtA: central artery, CYP: cytochrome p450, DMSO: dimethylsulfoxide, hpf: hours post fertilization, ISH: *in situ* hybridization, PCS: posterior communicating segments, PHBC: primordial hindbrain channel, WT: wildtype.

AHR signaling activates expression of CYP1 genes. We therefore analyzed the endogenous and BNF-induced expression of *cyp1a1/b1* in order to understand where AHR signaling might be activated, paying particular attention to vascular-specific domains. Both *cyp1a1* and *cyp1b1* were strongly upregulated in embryos exposed to BNF (**Fig 2A and 2B**). In the trunk neither enzyme showed strong endogenous expression in the epidermis, and *cyp1a1* was the only gene detected in the vasculature (**S1A and S1B Fig**). Specifically, we observed strong expression in the PCV, intersegmental vessels (ISVs), and DLAV, with limited expression in a few cells of the DA. BNF led to high induction of *cyp1a1* expression in the epidermis, precluding imaging of blood vessels located more deeply within the tissue (**S1C Fig**). *cyp1b1* expression was induced in the epidermis to a lower extent by BNF, permitting the visualization of internal structures. We detected induced *cyp1b1* expression in the DA, DLAV and ISVs of the trunk (**S1D Fig**). Thus, *cyp1a1* is the predominate CYP gene expressed in the trunk vasculature and is highly enriched in the vein and small vessels with less expression in the primary artery.

We next asked if endogenous CYP1 expression might be similarly distributed between arteries, veins and capillaries in other vascular beds and turned our attention to the zebrafish cranial vasculature (**Fig 2C**). At this stage, the hindbrain vasculature is composed of two bilaterally paired veins, the primordial hindbrain channels (PHBCs). Between them lies the primary arterial input into the brain, the basilar artery (BA), branching via the paired posterior communicating segments (PCS) to form the basal communicating artery (BCA). The central arteries (CtAs) extend dorsally and medially to connect the PHBCs to the BA and irrigate the neuronal tissue. In the plane below these brain vessels the bifurcated lateral dorsal aortae (LDA) collect blood from the heart to be delivered posteriorly toward the trunk via the DA (**S1E Fig**).

We found *cyp1a1* to be restricted to cranial arteries, including the BA, BCA, and PCS **(Fig 2D)**. While high expression was detected in the liver and the LDA (asterisk in **S1F Fig**), *cyp1a1* was notably excluded from the CtAs and PHBCs **(Fig 2D–2F)**. In the anterior head, we also detected *cyp1a1* in the paired ventral primitive internal carotid arteries (PICA) between the eyes, and in the optic artery (OA) (**S1G and S1H Fig**). Additionally, *cyp1a1* was highly expressed in the outer eye epithelium but not in the optic furrow (**S1I Fig**, arrow)

In comparison to *cyp1a1*, *cyp1b1* displayed a more narrow expression pattern and was detected in the developing ear and a region in the middle of the brain, but was not detectable in any vascular structures (**Fig 2G–2I**). This finding extended to the anterior head as well, where *cyp1b1* expression was absent from the liver as well as the LDA, PICA and OA vessels (**S1J–S1L Fig**). Therefore, as in the trunk, the vascular structures of the head are normally devoid of *cyp1b1* expression. Interestingly, in the eye, *cyp1b1* was primarily expressed near the base of and in a swath along the optic furrow (**S1M Fig,** arrow). Therefore, these two CYP genes display a complementary pattern of expression in the embryonic eye, with *cyp1b1* expression covering those optic regions lacking *cyp1a1*.

Lastly, we analyzed BNF-induced changes in CYP1 expression. As already shown, BNF upregulates *cyp1a1* to such an extent as to make imaging of interior structures impossible with conventional light microscopy (**Fig 2B**). We therefore leveraged a previously published *ahr2* mutant [29] to examine BNF-induced *cyp1a1* expression in the cranial vasculature. This mutant fails to induce *cyp1a1* in the skin, enabling the visualization of interior vessels. *cyp1a1* expression was preserved in the BA, PCS and BCA, but BNF treatment expanded its expression into the PHBC and CtAs as well, rendering all brain vessels *cyp1a1*-positive (**Fig 2J–2L**). In the anterior head, *ahr2* mutants had *cyp1a1* expression in the liver, LDA and PICA vessels, and strong upregulation in the eye (**S1N–S1Q Fig**). In wildtype embryos, we found that BNF induced the expression of *cyp1b1* in the LDA, but did not cause an upregulation in the liver (**S1R Fig**). Strikingly, in the hindbrain BNF treatment induced *cyp1b1* expression only in a subset of vessels. Expression was readily detected in the PHBC, but not the BCA, BA or PCS (**Fig 2M**). *cyp1b1* expression was induced in the CtAs as well, but in a somewhat more punctate fashion than *cyp1a1* (compare **Fig 2K** to **2N**). Therefore, in the hindbrain *cyp1b1* induction is excluded in those vessels that are highly *cyp1a1*-positive under normal conditions (**Fig 2O**). In the anterior head, *cyp1b1* induction was observed in the PICA vessels, but not the OA (**S1S and S1T Fig**). In the eye, the induction of epidermal *cyp1b1* expression by BNF in addition to the basal domain in the optic furrow was also observed (**S1U Fig,** compare to **S1M**). Thus, while *cyp1a1* showed basal levels of expression within endothelial cells, we detected *cyp1b1* expression within blood vessels only after AHR agonist treatment. The marked induction of both *cyp1a1/b1* in the CtAs, which are normally negative for either CYP gene, prompted us to examine if the CtAs had an otherwise normal gene expression profile, and we therefore analyzed *glut1* expression in BNF treated embryos. This gene encodes a glucose transporter integral for blood-brain-barrier (BBB) formation known to be enriched in the zebrafish brain [37]. We found that vascular *glut1* expression was restricted to brain capillaries including the CtAs, and this expression was reduced by BNF treatment (**S2 Fig**), indicating that BNF-induced AHR activation might affect BBB maturation.

These findings illustrate that, similarly to aryl hydrocarbon receptors, CYP function in the embryo appears partitioned to different genes with different expression domains that complement one another. This is tightly regulated such that the brain arteries (but not capillaries or veins) are *cyp1a1*-positive in contrast to the trunk vasculature, which has high *cyp1a1* expression in the vein and capillaries but less in the primary artery. Interestingly, hyper-activation of the AHR pathway by BNF induces *cyp1b1* in blood vessels that ordinarily do not express high levels of either CYP gene (e.g. the PHBCs and CtAs).

### Generation of zebrafish *ahr1a* and *ahr1b* mutants

**Fig 3:**
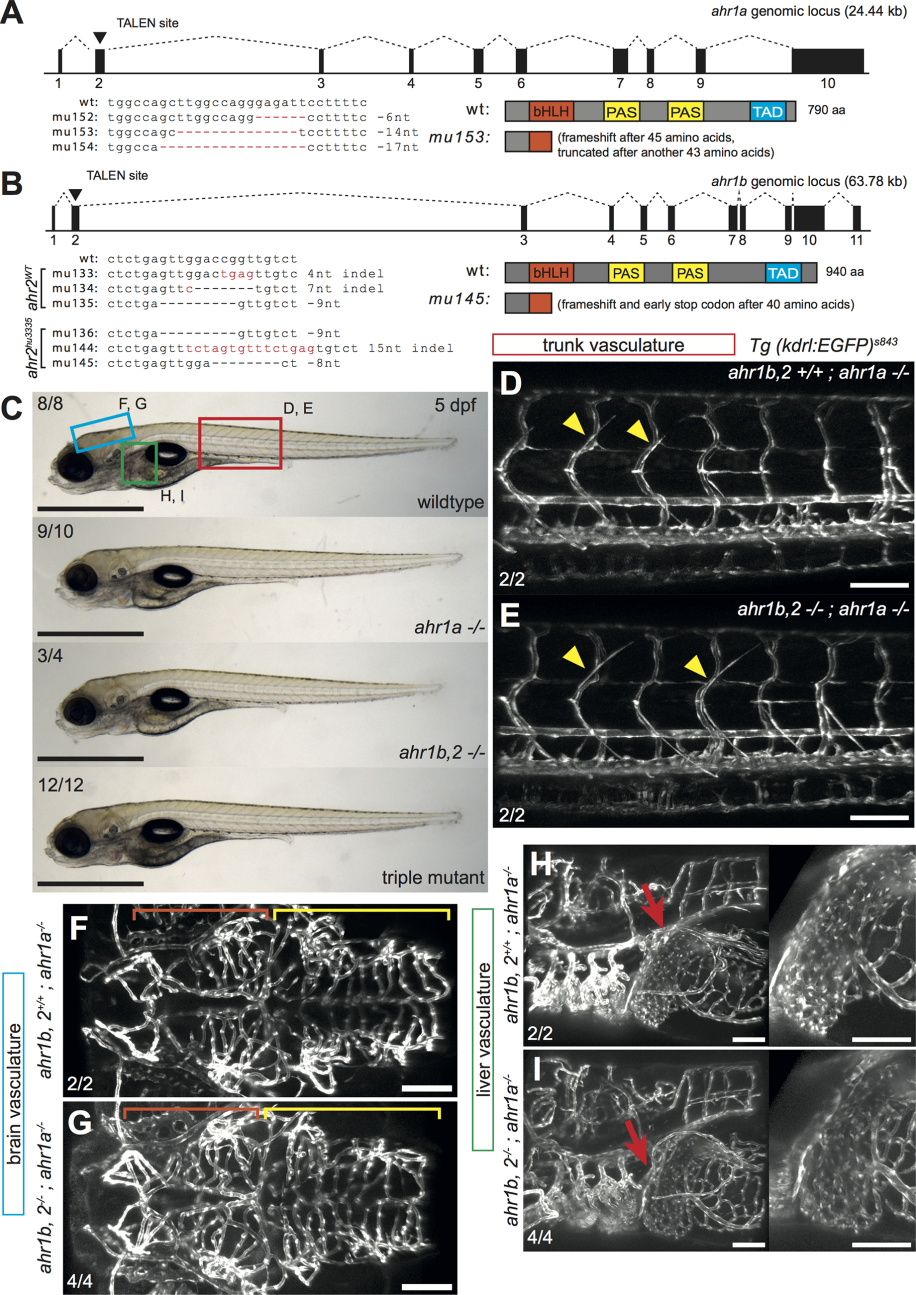
Generation of zebrafish *ahr1a* and *ahr1b* mutants. **(A, B)** Depiction of genomic locus for *ahr1a* (**A**) and *ahr1b* (**B**). TALEN pairs were designed targeting the bHLH domain in exon 2 of both genes. Genomic lesions in the TALEN spacer indicated in alignment to WT sequence, and cartoons show predicted protein products. For *ahr1b*, alleles mu133-mu135 were generated in a WT genetic background, while mu136-mu145 were made in an *ahr2*^*hu3335*^ homozygous mutant background. (**C)** Brightfield images of WT, *ahr1a* -/-, *ahr1b*,*2 -/-* and triple AHR mutant embryos at 5 dpf. No gross morphological defects in AHR mutants are apparent. Boxes indicate region of imaging for different vascular beds in **D-I**. Numbers correspond to “number of embryos with inflated swim bladders/total number of embryos of that genotype”. Scale bar is 1000 um. (**D-I)** Maximum intensity projections of confocal z-stacks comparing the trunk (**D, E**), brain (**F, G**) and liver (**H, I**) vascular beds in *ahr1a* -/- and triple AHR mutant zebrafish embryos at 5 dpf. Vascular patterning appears normal in both mutants compared to WT. Yellow arrowheads point to forming intercostal vessels in the trunk. Orange brackets mark midbrain vessels, yellow brackets mark hindbrain vessels. Red arrows show the liver. Numbers indicate the “number of embryos of a particular genotype with vascular characteristics as in image/total numbers of that genotype analyzed.” Scale bar is 100 um. Abbreviations—AHR: aryl hydrocarbon receptor, bHLH: basic helix-loop-helix, hpf: hours post fertilization, TALEN: transcription activator-like effector nuclease, WT: wildtype.

To study the function of AHR in vascular development, we generated zebrafish mutants that lack all AHR function. While a TILLING allele (hu3335) in *ahr2* [29] was readily available, we used TALEN pairs targeted to a region in the bHLH domain of exon 2 in both *ahr1b* and *ahr1a* to generate further mutants (**Fig 3A and 3B**). Importantly, since *ahr1b* and *ahr2* are tightly linked, we obtained *ahr1b* mutants in WT (mu133-mu135) and *ahr2*^*hu3335/hu3335*^(mu136, mu144, mu145) genetic backgrounds to ensure that we could study the function of *ahr1b* alone in single-mutants or in *ahr1b*/*ahr2*^*hu3335*^ double mutants (hereafter written as *ahr1b*,*2 -/-*) (**Fig 3B**). We chose the *ahr1a*^*mu153*^ and *ahr1b*^*mu145*^ alleles for subsequent analysis because both consist of even-numbered nucleotide deletions that lead to predicted frameshifts and early stop codons, and together allow for the study of triple AHR mutant embryos. To our surprise, *ahr1a* -/- and triple AHR-deficient embryos were viable and morphologically indistinguishable from their WT siblings, forming normal swim bladders and feeding by 5dpf (**Fig 3C**). We analyzed a number of vascular beds at different developmental stages, but did not observe obvious patterning defects. Angiogenesis in the trunk proceeded normally, and triple mutants developed the full complement of ISVs and had circulation (**Fig 3D and 3E**). The brain had no overt vascular defects, and both midbrain and hindbrain compartments were well vascularized (**Fig 3F and 3G**). Due to the reported liver vascular defects in AHR -/- mice [12], we examined the liver vasculature in our mutants at 5 dpf (**Fig 3H and 3I**). However, there were no gross differences in size and complexity and no obvious evidence of shunting, suggesting that AHR is dispensable for embryonic vascular patterning in zebrafish.

### Dissection of AHR-dependent and independent control of CYP1 expression

We considered the following 3 possibilities to explain the lack of detectable vascular phenotypes in triple AHR mutant embryos:

- no requirement for AHR during vascular development in zebrafish
- as yet unnoticed subtle defect(s) in specific vascular beds or specific developmental stages
- AHR alleles generated by TALEN mutagenesis are not loss of function

**Fig 4:**
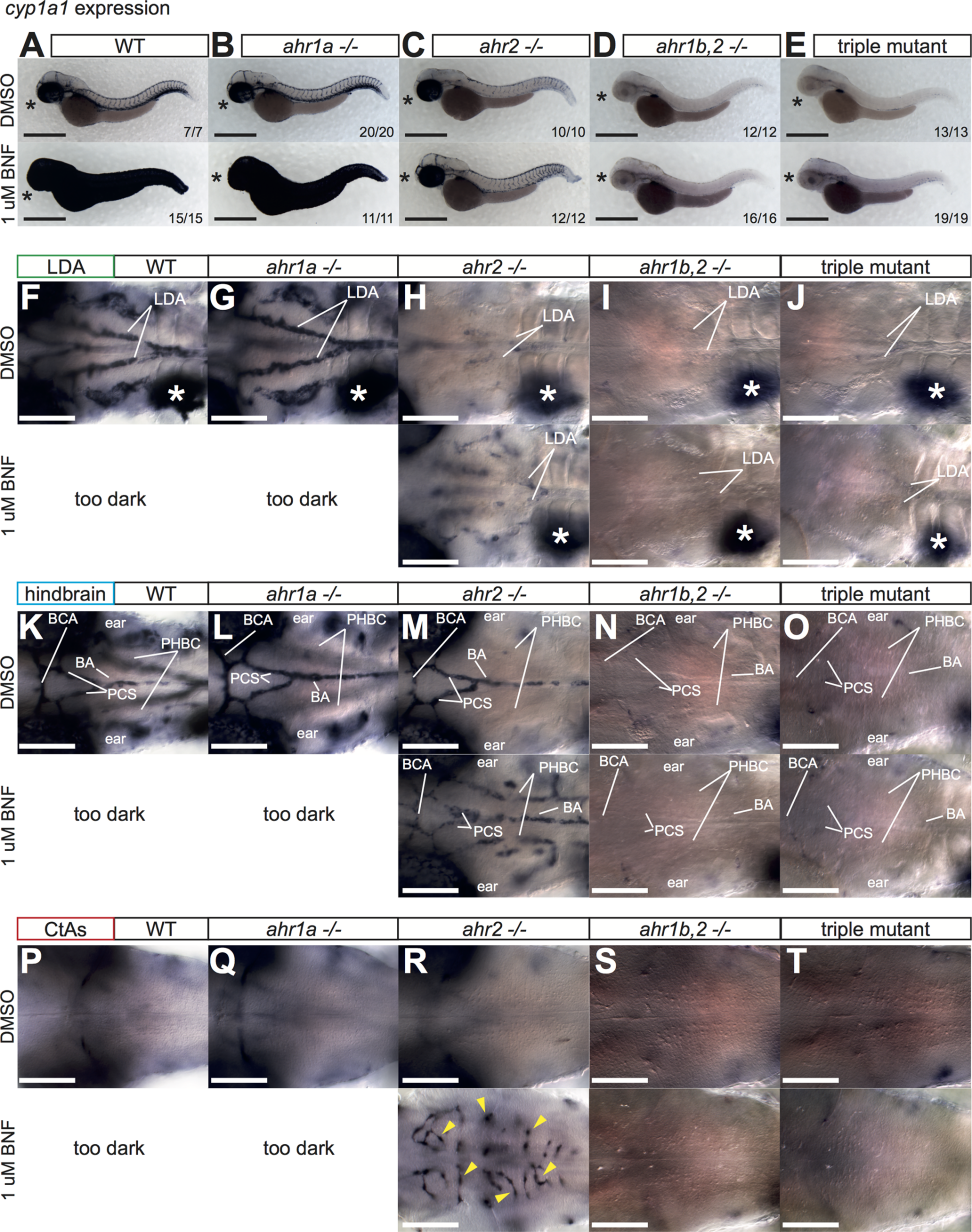
Regulation of *cyp1a1* by AHR. **(A)** Overview pictures of *cyp1a1* expression at 52 hpf in DMSO or BNF-treated WT (**A**), *ahr1a -/-* (**B**), *ahr2 -/-* (**C**), *ahr1b*,*2 -/-* **(D**) and triple AHR mutants (**E**). *ahr1a -/-* are indistinguishable from WT. (*) indicates expression in the eye in DMSO and BNF-treated embryos, which is lost only in *ahr1b*,*2 -/-* and triple AHR mutants. Note reduction of endogenous vascular *cyp1a1* expression in *ahr2* mutants (**C,** upper panels) that is lost in *ahr1b*,*2 -/-* and triple AHR mutants (**D, E** upper panels). *ahr2 -/-* embryos treated with BNF do not induce *cyp1a1* expression in the skin, revealing staining in blood vessels underneath (**C**, lower panel), which is abolished in *ahr1b*,*2 -/-* and triple AHR mutants (**D, E**, lower panels). Scale bar is 500 um. (**F-T)** High magnification images of blood vessels of 3 different planes of the head vasculature: the LDA (**F-J**), the hindbrain blood vessels (**K-O**) and the hindbrain capillaries (**P-T**) in all genetic combinations with DMSO and BNF treatment. Note strong dependence of LDA (but not hindbrain arteries, PHBC or CtAs) on *ahr2* for full *cyp1a1* expression. White asterisk denotes liver. Yellow arrowheads label individual CtAs. Numbers indicate embryos with indicated expression pattern/total embryos of that genotype analyzed. Scale bar is 100 um. Abbreviations–AHR: aryl hydrocarbon receptor, BNF: beta-naphthoflavone, CtA: central artery, CYP: cytochrome p450, DMSO: dimethylsulfoxide, hpf: hours post fertilization, LDA: lateral dorsal aortae, PHBC: primordial hindbrain channel, WT: wildtype.

As CYP1 expression is routinely used as a proxy for active AHR signaling, we employed the previous BNF/CYP1 assay with our newly generated mutants to determine if our alleles were indeed nonfunctional, and unravel the contribution of individual AHRs to endogenous and pharmacologically-induced CYP1 expression, starting with *cyp1a1*. Initial experiments showed no differences in *cyp1a1* expression in *ahr1b -/-* embryos compared to WT, and we therefore focused our analysis on embryos obtained from in-crosses of *ahr1a*^*mu153*^ +/-; *ahr1b*^*mu145*^ +/-; *ahr2*^*hu3335*^ +/- adults, yielding clutches containing WT, *ahr1a -/-*, *ahr1b*,*2 -/-* and triple AHR mutant embryos. We found no difference between WT and single *ahr1a* mutants (**Fig 4A and 4B**) in terms of endogenous or BNF-induced *cyp1a1* expression. *ahr2* mutants maintained high levels of *cyp1a1* in the eye, but had clearly reduced endogenous expression of *cyp1a1* in the trunk vasculature (**Fig 4C,** upper panel). Closer examination of the trunk revealed that in *ahr2* mutants, endogenous *cyp1a1* expression was almost completely lost in the DA and PCV vessels compared to WT and *ahr1a -/-* mutants, while somewhat reduced *cyp1a1* levels persisted in the ISVs and DLAV (**S3A–S3C Fig**, upper panels). This remaining vascular expression could be augmented by BNF treatment, observable owing to the aforementioned loss of *cyp1a1* induction in the skin of *ahr2* mutants (**Fig 4C** and **S3C Fig**, lower panels). Finally, analysis of *ahr1b*,*2* and triple AHR mutants showed loss of all *cyp1a1* expression in the eye and in the trunk blood vessels, while gut and liver expression were unaffected (**Fig 4D and 4E**, and **S3D and S3E Fig**, lower panels). These results demonstrate firstly that *ahr1b*^*mu145*^ is very likely a loss of function allele, and that *ahr1b* can functionally compensate for loss of *ahr2* in the eye and in some, but not all, zebrafish blood vessels. Secondly, they also indicate that not all *cyp1a1* expression is dependent on AHR.

To address whether functional overlap exists between *ahr1b* and *ahr2* in other vessels, we extended our analysis of *cyp1a1* expression to the cranial vasculature in different AHR mutants. In the LDA, *cyp1a1* expression was normal in *ahr1a* mutants, almost completely abrogated in *ahr2* mutants and lost in *ahr1b*,*2* and triple AHR-deficient embryos (**Fig 4F–4J**, upper panels). *cyp1a1* could be detected in the liver for all genetic combinations of AHR alleles. BNF could weakly stimulate *cyp1a1* in the LDA of *ahr2* mutants, but not double *ahr1b*,*2* and triple AHR mutants (**Fig 4H–4J**, lower panels). Analysis of the hindbrain revealed that the endogenous *cyp1a1* expression in the BA, BCA and PCS vessels was refractory to loss of *ahr1a* and *ahr2*, and was only eliminated in *ahr1b*,*2* and triple AHR mutants (**Fig 4K–4O**, upper panels). The induction of *cyp1a1* in the PHBC vessels by BNF previously observed in *ahr2* mutants did not occur in double- and triple AHR fish (**Fig 4M–4O,** lower panels). Similarly, *cyp1a1* induction by BNF in the normally *cyp1a1*-negative CtAs persisted in *ahr2* mutants, and was abolished in *ahr1b*,*2* and triple AHR mutants (**Fig 4P–4T**).

Together, these results highlight a differential capacity for *ahr1b* to compensate for *ahr2* in controlling *cyp1a1* expression in embryonic vessels: strong *ahr1b* activity in the ISVs, DLAV, BA, PCS, PHBCs and CtAs, with minimal contribution to *cyp1a1* expression in the LDA, DA and PCV.

**Fig 5:**
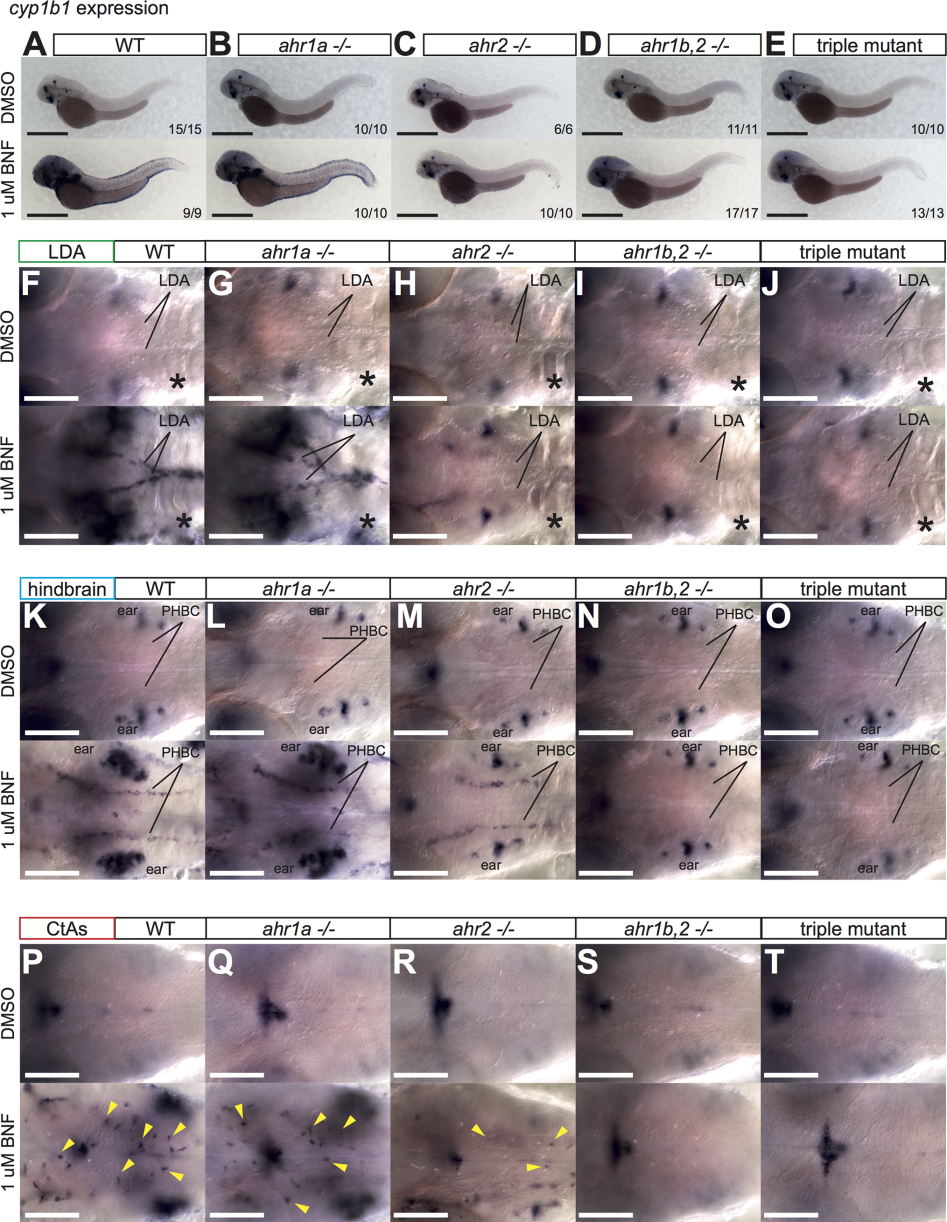
Regulation of *cyp1b1* by AHR. **(A-E)** Overview pictures of *cyp1b1* expression at 52 hpf in DMSO or BNF-treated WT (**A**), *ahr1a -/-* (**B**), *ahr2 -/-* (**C**), *ahr1b*,*2 -/-* **(D**) and triple AHR mutants (**E**). No changes in expression pattern were evident in DMSO-treated embryos that lacked any AHR genes. Induction in the skin by BNF was dramatically reduced in *ahr2 -/-* (compare **A** to **C**, lower panels). Scale bar is 500 um. **F-T)** High magnification images of blood vessels in 3 different planes of the head vasculature: the LDA (**F-J**), the hindbrain blood vessels (**K-O**) and the hindbrain capillaries (**P-T**) in all genetic combinations with DMSO and BNF treatment. Black asterisks denote approximate location of liver. Yellow arrowheads label individual CtAs. Note maintenance of endogenous *cyp1b1* expression in the ear (**K-O**, upper panels) and head (**P-T**, upper panels) of all mutants. Expression of *cyp1b1* was not detected in the liver for either treatment in any genetic combination (**F-J**). Note lack of BNF-induced staining in LDA of *ahr2* mutants, but strong staining in PHBCs (**H** and **M,** lower panels). Numbers indicate embryos with indicated expression pattern/total embryos of that genotype analyzed. Scale bar is 100 um. Abbreviations–AHR: aryl hydrocarbon receptor, BNF: beta-naphthoflavone, CtA: central artery, CYP: cytochrome p450, DMSO: dimethylsulfoxide, hpf: hours post fertilization, LDA: lateral dorsal aortae, PHBC: primordial hindbrain channel, WT: wildtype.

Analysis of *cyp1b1* expression showed no effect of loss of any or all AHR gene function on the endogenous levels of *cyp1b1* (**Fig 5A–5E**, upper panels). Both WT and *ahr1a* mutants displayed high levels of *cyp1b1* in the skin in response to BNF, which did not occur in *ahr2*, *ahr1b*,*2* and triple AHR mutants (**Fig 5A–5E**, lower panels). In the trunk, the vascular *cyp1b1* expression induced by BNF weakly persisted in the ISVs and DLAV of *ahr2* mutants and was absent from *ahr1b*,*2* and triple AHR mutants, mirroring the results we obtained for *cyp1a1*, namely that *ahr1b* is functional in the DLAV and ISVs and less in the DA and PCV (**S3F–S3J Fig**). Similarly, analysis of the cranial vessels showed no induction of *cyp1b1* in the LDA of *ahr2* mutants, while induction in the CtAs and PHBC could occur in the absence of *ahr2*, but not in embryos lacking *ahr1b*,*2* or all 3 AHRs (**Fig 5F–5T**). These results highlight the importance of both *ahr1b* and *ahr2* in zebrafish endothelium, and emphasize the nuanced spatial requirements for AHR function across the embryo in physiologic and toxicological responses.

### Transcriptional response of the chemokine receptor *cxcr4a* to BNF is AHR-dependent

**Fig 6:**
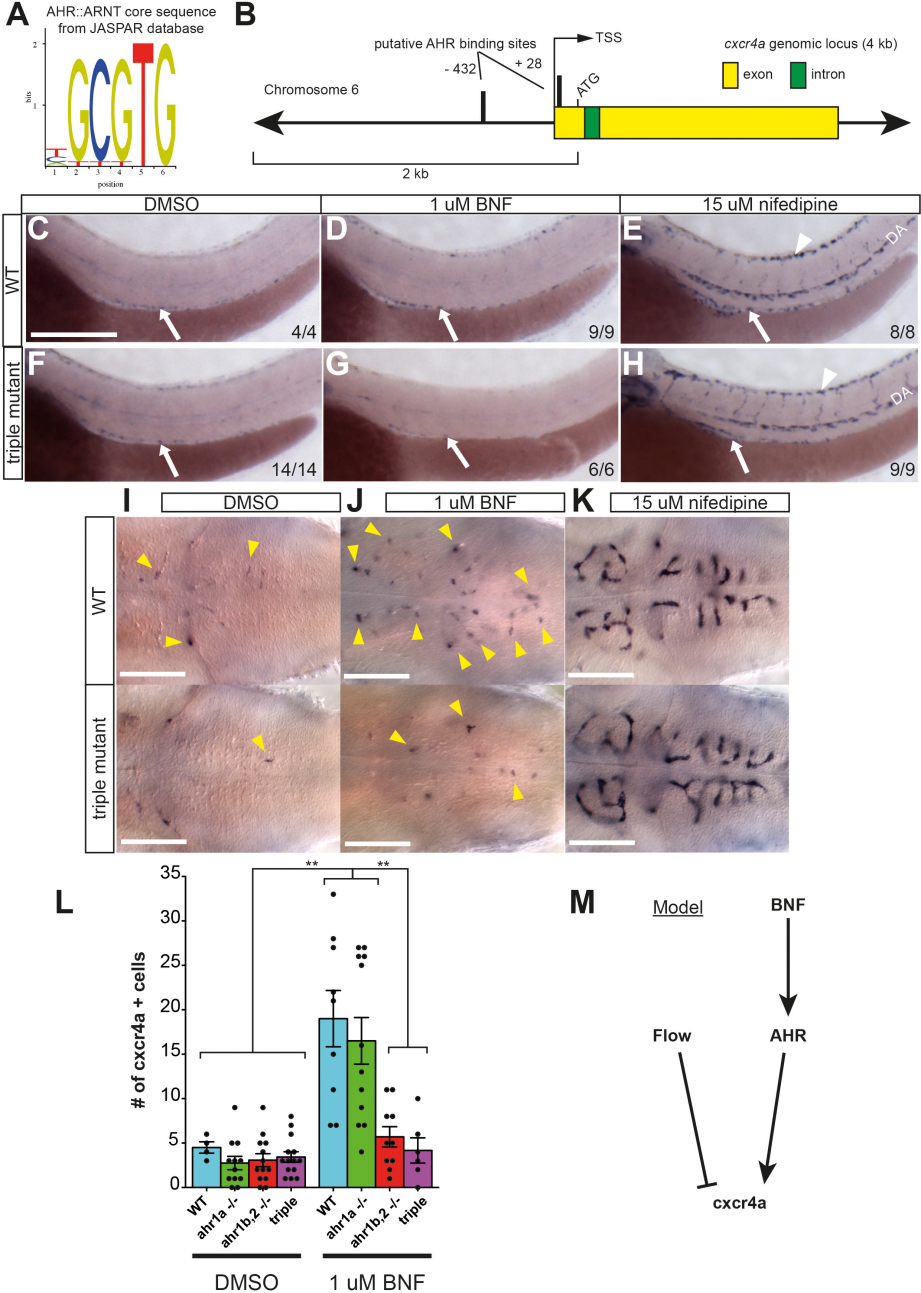
Transcriptional control of *cxcr4a* by AHR. **(A)** Core DNA binding sequence of AHR::ARNT complex from JASPAR database. (**B)** Schematic of the zebrafish *cxcr4a* gene. Using strict search criteria (a relative profile score threshold of 90%), the promoter sequence spanning 2 kb from the start codon was analyzed for AHR binding sites in the JASPAR database. Two putative AHR binding sites lie close to the transcription start site (TSS). (**C-H)** Whole mount ISH for *cxcr4a* in WT and triple AHR mutant embryos treated from 48 hpf to 52 hpf with DMSO (**C, F**), 1 uM BNF (**D, G**) or 15 uM nifedipine (**E, H**). Arrows indicate subintestinal vasculature, while arrowheads point to DLAV. Scale bar is 250 um. (**I-K**) High magnification images of hindbrain capillaries in WTs and triple AHR mutants. Note similar levels of *cxcr4a*-positive CtAs (yellow arrowheads) in WTs and triple AHR mutants treated with DMSO (**I**), and markedly fewer *cxcr4a*-positive cells in triple mutants treated with BNF compared to WT (**J**). Both WTs and triple AHR mutants display marked increase in *cxcr4a* expression in all CtA endothelial cells after nifedipine treatment (**K**). Scale bar is 100 um. (**L)** Quantification of *cxcr4a*-positive endothelial cells in high magnification images of the hindbrain of WT, *ahr1a -/-*, *ahr1b*,*2 -/-* and triple AHR mutants treated from 48–52 hpf with DMSO or nifedipine. All genetic combinations of AHR mutations have similar numbers of *cxcr4a*-positive cells at basal levels. The BNF-induced increase of *cxcr4a* expression in WT still occurs in *ahr1a -/-* embryos, but does not show in *ahr1b*,*2 -/-* or triple AHR mutants. Analyzed by One-way ANOVA (DMSO treatment N = 4 WT, 12 *ahr1a -/-*, 13 *ahr1b*,*2 -/-* and 14 triple AHR fish. 1 uM BNF treatment N = 9 WT, 12 *ahr1a -/-*, 10 *ahr1b*,*2 -/-* and 6 triple AHR fish.) (**M)** Summary of transcriptional control of *cxcr4a* expression by flow (negative regulator) and BNF (positive regulator). Induction by BNF is in an AHR-dependent manner. **P<0.01, error bars indicate s.e.m. Abbreviations–AHR: aryl hydrocarbon receptor, ARNT: aryl hydrocarbon nuclear translocator, BNF: beta-naphthoflavone, CtA: central artery, CYP: cytochrome p450, DLAV: dorsal longitudinal anastomotic vessel, DMSO: dimethylsulfoxide, hpf: hours post fertilization, ISH: *in situ* hybridization, LDA: lateral dorsal aortae, PHBC: primordial hindbrain channel, TSS: transcription start site, WT: wildtype.

The chemokine receptor *cxcr4a* has been reported to be differentially expressed in zebrafish embryos exposed to AHR ligands [38]. However, this study was performed by microarray analysis of whole embryo lysate, and therefore no spatial information on the nature of this regulation is known. The AHR::ARNT heterodimer binds a canonical AHR response element, (TNGCGTG) (**Fig 6A**) [39]. Using the 2 kb promoter sequence 5’ of the ATG start codon in *cxcr4a* as a template, a search of the JASPAR online database of transcription factor binding motifs revealed 2 putative AHR binding sites (**Fig 6B**). To test the hypothesis that AHR may be a transcription factor capable of regulating *cxcr4a* expression, we treated WT embryos from 48–52 hpf with DMSO or 1 uM BNF and performed an ISH for *cxcr4a*. At this stage, vascular *cxcr4a* expression was essentially limited to the aortic arch vessels (not shown), the forming subintestinal vasculature (**Fig 6C,** arrow) and a few hindbrain CtAs (**Fig 6I**, arrowheads). Interestingly, BNF treatment led to an increased expression of *cxcr4a* specifically in endothelial cells, such as the subintestinal vasculature (**Fig 6D**, arrow), and particularly in the hindbrain (**Fig 6J**, arrowheads). We next asked if this induction was similar to the upregulation of *cxcr4a* known to occur in embryos without blood flow [40]. Nifedipine is a calcium channel blocker, and its administration to embryos stops the heartbeat, causing blood flow to cease. This leads to dramatic upregulation of *cxcr4a* in the DA (**Fig 6E**) and DLAV (**Fig 6E**, arrowhead) of the trunk and in the cranial vessels (**Fig 6K**). This response was stronger than that induced by BNF, and seemed to occur in more ECs of the blood vessels. To determine if AHR was responsible for any endogenous or pharmacologically-induced *cxcr4a* expression, we analyzed triple AHR mutant embryos. Basal expression in the subintestinal vasculature was unaltered in triple AHR mutants treated with DMSO (asterisk in **Fig 6F**, arrow). However, the BNF-induced increase in *cxcr4a* expression in the hindbrain was attenuated (**Fig 6J**, arrowheads). The response to flow block appeared unchanged, and we observed strong induction of *cxcr4a* in the trunk and head vessels to the same extent as WT (**Fig 6H** and **6K**). Due to the rather robust upregulation of *cxcr4a* in the CtAs under BNF treatment, we turned to this vascular bed to analyze *cxcr4a* regulation by BNF in AHR mutants in more detail. We counted the number of *cxcr4a*-positive cells in WT, *ahr1a -/-*, *ahr1b*,*2* -/- and triple AHR mutant embryos treated with DMSO and BNF. The endogenous expression of *cxcr4a* appeared unaffected by loss of any AHR genes, whereas the BNF-induced increase in expression was abrogated in *ahr1b*,*2* -/- and triple AHR mutants compared to WT and *ahr1a -/-* (**Fig 6L**). Our results therefore lead to a model where flow and AHR have opposing and independent effects on *cxcr4a* transcription. Ectopic activation of AHR by BNF enhances the expression of *cxcr4a* in endothelial cells, whereas blood flow serves to negatively regulate this chemokine receptor, and AHR is not responsible for the increase in *cxcr4a* expression when blood flow is compromised (**Fig 6M**).

## Discussion

### Generation of AHR-deficient zebrafish

Studying AHR function in fish is challenging based simply on the number of AHR family genes in their genomes. This work is the first to examine the effect of genetic deletion of all AHR gene products in the zebrafish, and to correlate their respective contributions to AHR signaling in different tissues. In line with published expression data, we observed broad embryonic expression of *ahr2*, including the endoderm, skin and close to the trunk axial vessels [23]. We show that the other two zebrafish AHRs, *ahr1a* and *ahr1b*, display more restricted expression patterns, and we only detected them in the liver and eye, respectively. We could demonstrate functional redundancy of both *ahr1b* and *ahr2* in the eye, where their overlapping expression ensures *cyp1a1* transcription in *ahr1b*- or *ahr2*-single but not double mutants. The transcription of zebrafish AHRs responds differentially to the agonist BNF, which induces *ahr2* and *ahr1a* but not *ahr1b* expression.

Our gain of function experiments with BNF confirmed previous findings that *ahr2* is responsible for most of the ligand-induced *cyp1a1/cyp1b1* expression in zebrafish [25, 29]. However, these studies reported only qPCR data on CYP transcripts in whole embryo lysate (no spatial information) or only focused on the effect of *ahr2* knockdown on induced CYP levels (little attention to endogenous CYP transcript with knockdown or knockout of *ahr2*). Our analysis of different vascular beds revealed distinct requirements for *ahr1b* and *ahr2* in specific vessels. *ahr1b* is required to maintain basal *cyp1a1* expression in the arteries of the hindbrain and the ISVs of the trunk in the absence of *ahr2*. Surprisingly, this compensation does not occur in all vessels, and we observed that the DA, PCV and LDA were highly dependent on *ahr2* for full *cyp1a1* expression. By amplifying AHR activity with BNF, we could demonstrate overlapping function of *ahr1b* and *ahr2* in inducing *cyp1a1* and *cyp1b1* in the PHBCs and CtAs of the hindbrain, vessels that are normally negative for both CYP1 genes. Our analysis of triple AHR mutants also demonstrates that endogenous *cyp1b1* expression and a baseline level of *cyp1a1* expression in the liver are AHR independent.

The spatial differences in vascular CYP1 expression and differential reliance on *ahr1b* and *ahr2* are striking. Why, for example, is the primary axial vein highly *cyp1a1*-positive while the hindbrain veins do not express either CYP gene? Why does *ahr1b* maintain *cyp1a1* expression in the hindbrain arteries but not the LDA in *ahr2* mutants? Why does BNF fail to induce *cyp1b1* expression in the *cyp1a1*-positive hindbrain arteries? Possible explanations include differential expression of AHRs themselves. ISH may not be sensitive enough to detect low levels of AHR transcript, and it may be that *ahr1b* is lowly expressed in the BA, PCS, BCA, CtAs and ISVs but not in the LDA, DA and PCV, thereby determining the vessels in which compensation is possible. We also did not examine changes in the expression patterns of all the AHR genes in our genetic mutants, and cannot rule out the possibility that loss of one may induce compensatory upregulation of the others.

The combined effects of AHR and hemodynamics could further explain the unique CYP expression profile in distinct vessels. *cyp1a1* and *cyp1b1* are the two most strongly upregulated genes in HUVECs exposed to shear stress in vitro [14, 41]. Regulation of *cyp1a1* by shear stress was dependent on AHR binding sites in the rat *cyp1a1* promoter *in vitro* [15]. In that study, fluid shear stress also led to an increase in expression and nuclear translocation of AHR. We recently showed that the PCV in the trunk experiences considerable hemodynamic forces at this stage of development [42]. A differential hemodynamic profile could potentially explain the lack of *cyp1a1* expression in the hindbrain veins. It is worth noting that the loss of endogenous *cyp1a1* expression we observed in our triple AHR mutants is a striking finding, as these embryos have no obvious flow defect. It remains to be seen if any zebrafish AHRs are flow-regulated themselves, or if they control the flow-responsiveness of *cyp1a1 in vivo*. Interestingly, we did not detect any endothelial *cyp1b1* expression in baseline conditions. *cyp1b1* was shown to be one of the most highly expressed CYP enzymes in human brain microvessels from patient biopsies [43]. More recently it was shown that B-catenin positively regulates *cyp1b1*, but not *cyp1a1*, in endothelioma cell lines [44], and the authors concluded that the ability of *cyp1b1* to synthesize retinoic acid (from retinol) and 20-HETE (from arachnidonic acid) were essential in modulating BBB function. Furthermore, this study demonstrated that B-catenin was required for the AHR-dependent induction of *cyp1b1* expression by TCDD. This could explain our observations that BNF treatment upregulated *cyp1b1* in a restricted set of blood vessels in brain, specifically those that are *cyp1a1*-negative in endogenous situations. In support of this, Ziegler et al. found an inverse relationship between the levels of *cyp1a1* and *cyp1b1* expression with and without B-catenin function, suggesting their expression was mutually exclusive. Regarding BBB integrity, our findings that BNF induces the downregulation of *glut1* expression in brain capillaries may be an additional mode of AHR ligand-induced toxicity. Dioxin has been shown to reduce the amount of tight junction proteins Claudin and ZO-1 in endothelial cells in an *in vitro* BBB system [45], and it also causes a reduction in Glut1 expression when applied to mouse embryonic carcinoma cells [46]. Our data are consistent with these findings and provide further *in vivo* evidence that impaired BBB function may result from AHR hyper-activation.

### AHR and vascular patterning

Our failure to detect a vascular phenotype in triple AHR mutants is somewhat perplexing, given the observed vascular defects in liver, eye and kidney of AHR-deficient mice [12]. Triple AHR mutant zebrafish are viable, and we observed no obvious abnormalities in vascular patterning during embryonic development. This does not exclude subtle defects in distinct vessels, phenotypes relating to altered cell cycle progression or vascular tone for example, that might require careful quantification to resolve. We could show that the *ahr1b*^*mu145*^ allele is likely non-functional, and it is very probable that *ahr1a*^*mu153*^ is also a null allele, as it is targeted to the same bHLH region of the open reading frame. It has been reported that zebrafish *ahr1a* does not bind well to the AHR agonist TCDD or transcriptionally activate AHR luciferase reporters in *in vitro* experiments [22], and this led to the suggestion that *ahr1a* was an inactive pseudogene. However, more recent work suggested that induction of *cyp1a1* in the zebrafish liver in response to the compound leflunomide was *ahr1a*-dependent [29], and could indicate simply that *ahr1a* has higher affinity for different ligands. Application of leflunomide to the triple AHR mutants described here could provide evidence that the *ahr1a*^*mu153*^ allele is a functional null.

An interesting finding is the potential for AHR to transcriptionally regulate the expression of the chemokine receptor *cxcr4a* in response to exogenous ligands. While such regulation was reported in a microarray study [38], our study shows that this regulation occurs in endothelial cells. *cxcr4a* has been shown to be dramatically upregulated in zebrafish embryos lacking blood flow [40], and this hemodynamic regulation occurs normally in AHR mutants. Thus, our results show that flow and AHR have opposite effects on *cxcr4a* regulation, in contrast to their mutual positive effect on *cyp1a1* levels. At the time point of our analysis, we did not observe a change in endogenous *cxcr4a* expression in triple AHR mutants, nor did AHR-deficient embryos recapitulate the published vascular phenotypes seen in *cxcr4a*^*um20*^ mutants in LDA or hindbrain patterning [35, 40]. This indicates that AHR is dispensable for normal *cxcr4a* function during zebrafish embryonic development. Nevertheless, it is tempting to speculate that a link between AHR and *cxcr4* may be functionally relevant, as AHR -/- and CXCR4 -/- mice have remarkably similar vascular phenotypes in the kidney [12, 47]. Analysis of vascular remodeling at later stages of fish development may provide evidence of such a link.

## Supporting information

S1 Fig Endogenous and BNF-induced CYP1 expression in other vascular beds. **A-D)** High magnification images of *cyp1a1/b1* expression in trunk vasculature of DMSO (**A, B**) and BNF (**C, D**) treated embryos. Arrowheads point to ISVs. *cyp1a1* is expressed highly in PCV, ISVs and DLAV, and in isolated DA cells under normal conditions (**A**), and possible induction in blood vessels by BNF is obscured by high skin staining (**C**). No endogenous vascular *cyp1b1* can be detected (**B**), but it is induced in DA, DLAV and ISVs by BNF (**D**). **E**) *Camera lucida* drawing of 48 hpf zebrafish embryo from Kimmel, et al 1995 [36]. Dashed lines indicate plane of images in this figure to visualize lateral dorsal aortae (LDA, green). Confocal image shows dorsal view of LDA in live embryo at 48 hpf (scale bar is 200 um). Cartoon schematic depicts arrangement of LDA and DA. **F-M**) Cranial expression of *cyp1a1/b1* in DMSO-treated embryos. Expression of *cyp1a1* is mostly vascular-specific and restricted to arteries (LDA, PICA and OA). (*) marks the liver. No vascular or liver expression of *cyp1b1* is detected, and staining is only evident in the ear, middle of the brain and parts of the ventral eye. Arrow marks optic furrow. **N-U)** Cranial expression of *cyp1a1/b1* in BNF-treated embryos. In addition to the basal expression domains, specific blood vessels upregulate *cyp1b1* (the LDA and PICA). Note lack of *cyp1b1* expression in the liver even under BNF stimulation (* in **R**). Embryos in **N-Q** are *ahr2* mutants to enable imaging of interior vessels. Numbers of embryos analyzed are the same as in **Fig 2**. All scale bars are 100 um. Abbreviations–AHR: aryl hydrocarbon receptor, BNF: beta-naphthoflavone, CYP: cytochrome p450, DA: dorsal aorta, DLAV: dorsal longitudinal anastomotic vessel, DMSO: dimethylsulfoxide, hpf: hours post fertilization, ISV: intersegmental vessel, LDA: lateral dorsal aortae, OA: optic artery, PCV: posterior cardinal vein, PICA: primitive internal carotid artery.
(TIF)

S2 Fig Vascular *glut1* expression in WT embryos with and without BNF treatment. **A-C**) Overview and high magnification images of whole mount ISH for glut1 in WT embryos at 52 hpf. Vascular expression is limited to the brain capillaries (yellow arrowheads indicate individual CtAs). **D-F**) Glut1 expression in WT embryos treated with 1 uM BNF. Staining intensity is notably decreased in brain vessels. Scale bar in overview is 500 um, and 100 um in high magnification images. Abbreviations–BNF: beta-naphthoflavone, CtA: central artery, DMSO: dimethylsulfoxide, hpf: hours post fertilization, ISH: *in situ* hybridization.
(TIF)

S3 Fig Analysis of vascular CYP1 expression in trunk of AHR mutants. **A-J**) High magnification images of *cyp1a1/b1* expression in the trunk vasculature at 52 hpf in DMSO or BNF-treated WT (**A, F**), *ahr1a -/-* (**B, G**), *ahr2 -/-* (**C, H**), *ahr1b*,*2 -/-* **(D, I**) and triple AHR mutants (**E, J**). Expression patterns of both genes in *ahr1a -/-* are indistinguishable from WT in either condition. In *ahr2* mutants a dramatic loss of endogenous *cyp1a1* expression from the PCV and DA is observed, together with a weaker but persistent expression in DLAV and ISVs that is enhanced by BNF treatment (**C**). This remaining vascular expression is lost in *ahr1b*,*2 -/-* and triple AHR mutants (**D, E**). Note the AHR-independent expression of *cyp1a1* in the gut (arrows in **E**) Similar results are seen in the BNF-induced *cyp1b1* expression, which is weakly maintained in ISVs and DLAV of *ahr2* mutants and lost in *ahr1b*,*2 -/-* and triple AHR mutant embryos (**F-J**). Numbers of embryos analyzed are the same as in **Fig 4** (*cyp1a1*) and **Fig 5** (*cyp1b1*). All scale bars are 100 um. Abbreviations–AHR: aryl hydrocarbon receptor, BNF: beta-naphthoflavone, CYP: cytochrome p450, DA: dorsal aorta, DLAV: dorsal longitudinal anastomotic vessel, DMSO: dimethylsulfoxide, hpf: hours post fertilization, ISV: intersegmental vessel, PCV: posterior cardinal vein, WT: wildtype.
(TIF)
