# Supplementary figures and images for: Genetic dissection of endothelial transcriptional activity of zebrafish aryl hydrocarbon receptors (AHRs)
Source: PLoS One. 2017 Aug 17;12(8):e0183433. doi: 10.1371/journal.pone.0183433 (PMC5560736; doi:10.1371/journal.pone.0183433)

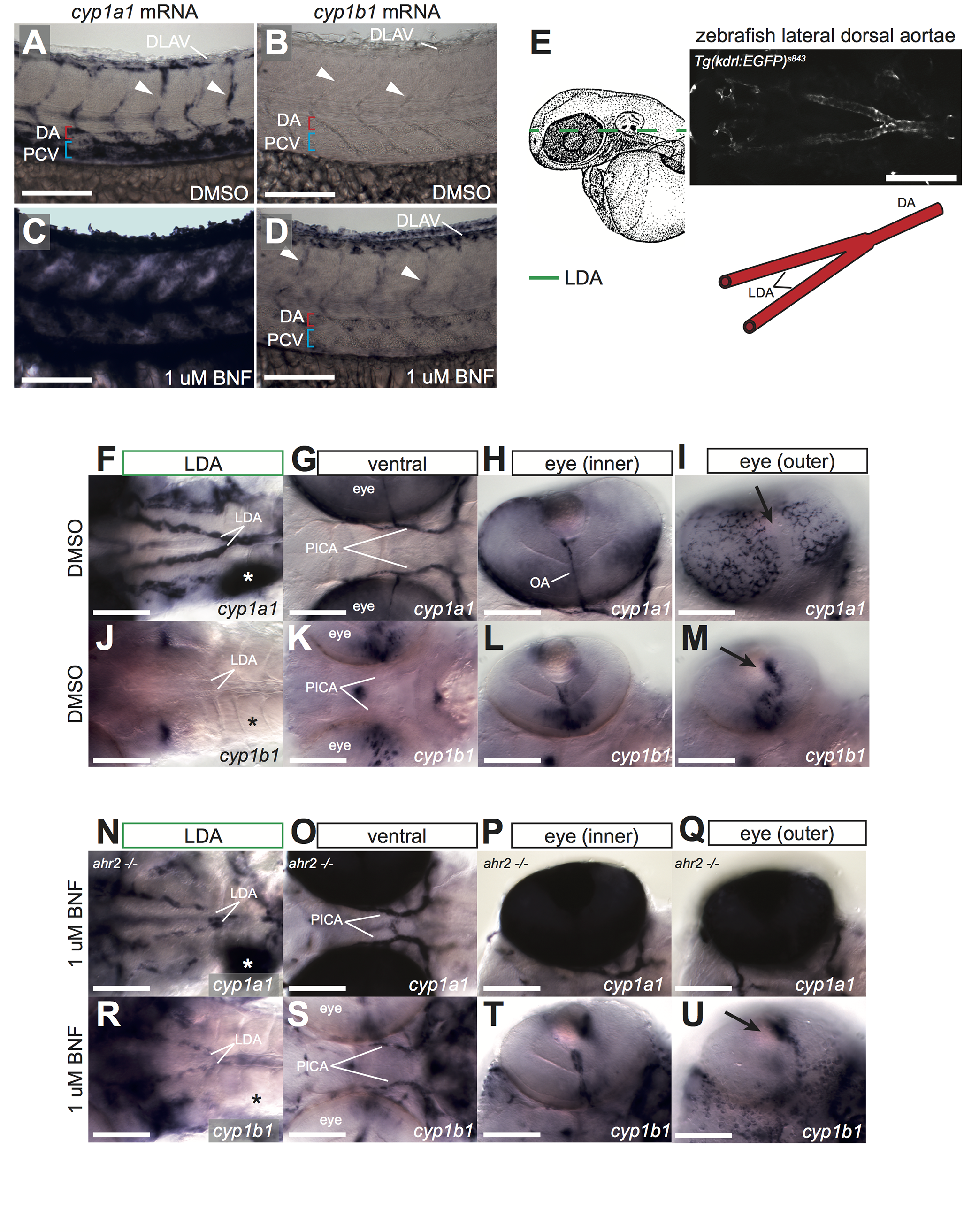

Supplement: S1 Fig — A-D) High magnification images of cyp1a1/b1 expression in trunk vasculature of DMSO (A, B) and BNF (C, D) treated embryos. Arrowheads point to ISVs. cyp1a1 is expressed highly in PCV, ISVs and DLAV, and in isolated DA cells under normal conditions (A), and possible induction in blood vessels by BNF is obscured by high skin staining (C). No endogenous vascular cyp1b1 can be detected (B), but it is induced in DA, DLAV and ISVs by BNF (D). E) Camera lucida drawing of 48 hpf zebrafish embryo from Kimmel, et al 1995 [36]. Dashed lines indicate plane of images in this figure to visualize lateral dorsal aortae (LDA, green). Confocal image shows dorsal view of LDA in live embryo at 48 hpf (scale bar is 200 um). Cartoon schematic depicts arrangement of LDA and DA. F-M) Cranial expression of cyp1a1/b1 in DMSO-treated embryos. Expression of cyp1a1 is mostly vascular-specific and restricted to arteries (LDA, PICA and OA). (*) marks the liver. No vascular or liver expression of cyp1b1 is detected, and staining is only evident in the ear, middle of the brain and parts of the ventral eye. Arrow marks optic furrow. N-U) Cranial expression of cyp1a1/b1 in BNF-treated embryos. In addition to the basal expression domains, specific blood vessels upregulate cyp1b1 (the LDA and PICA). Note lack of cyp1b1 expression in the liver even under BNF stimulation (* in R). Embryos in N-Q are ahr2 mutants to enable imaging of interior vessels. Numbers of embryos analyzed are the same as in Fig 2. All scale bars are 100 um. Abbreviations–AHR: aryl hydrocarbon receptor, BNF: beta-naphthoflavone, CYP: cytochrome p450, DA: dorsal aorta, DLAV: dorsal longitudinal anastomotic vessel, DMSO: dimethylsulfoxide, hpf: hours post fertilization, ISV: intersegmental vessel, LDA: lateral dorsal aortae, OA: optic artery, PCV: posterior cardinal vein, PICA: primitive internal carotid artery. (TIF) [file pone.0183433.s001.tif]

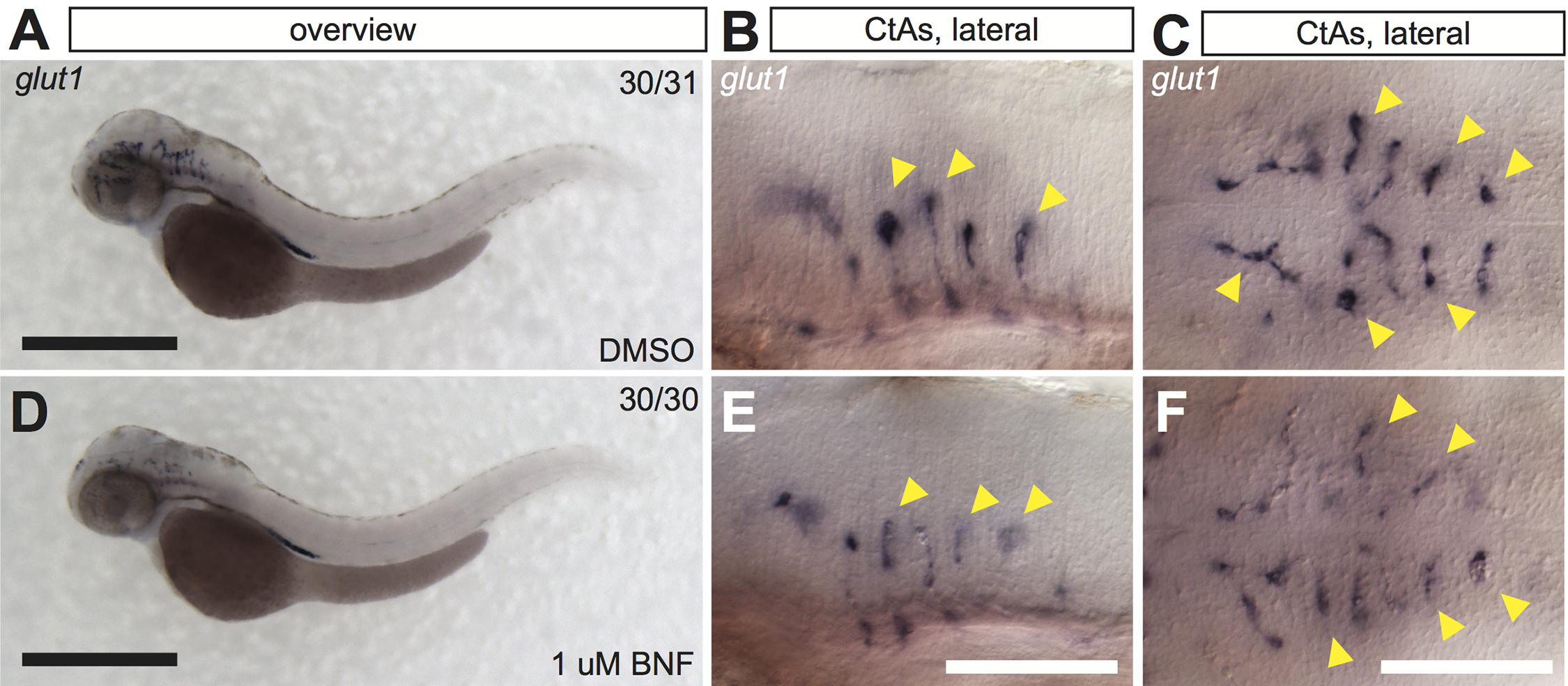

Supplement: S2 Fig — A-C) Overview and high magnification images of whole mount ISH for glut1 in WT embryos at 52 hpf. Vascular expression is limited to the brain capillaries (yellow arrowheads indicate individual CtAs). D-F) Glut1 expression in WT embryos treated with 1 uM BNF. Staining intensity is notably decreased in brain vessels. Scale bar in overview is 500 um, and 100 um in high magnification images. Abbreviations–BNF: beta-naphthoflavone, CtA: central artery, DMSO: dimethylsulfoxide, hpf: hours post fertilization, ISH: in situ hybridization. (TIF) [file pone.0183433.s002.tif]

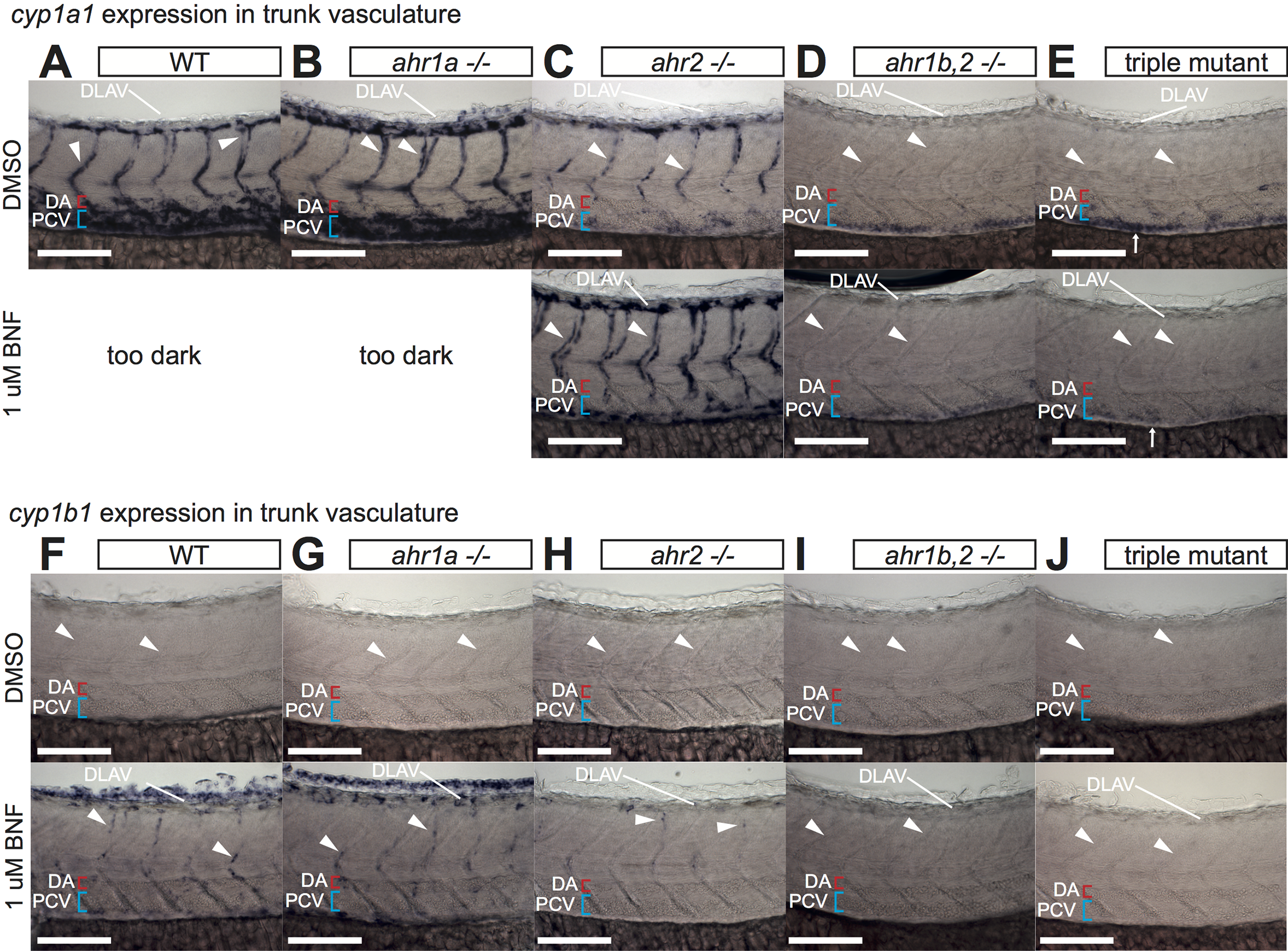

Supplement: S3 Fig — A-J) High magnification images of cyp1a1/b1 expression in the trunk vasculature at 52 hpf in DMSO or BNF-treated WT (A, F), ahr1a -/- (B, G), ahr2 -/- (C, H), ahr1b,2 -/- (D, I) and triple AHR mutants (E, J). Expression patterns of both genes in ahr1a -/- are indistinguishable from WT in either condition. In ahr2 mutants a dramatic loss of endogenous cyp1a1 expression from the PCV and DA is observed, together with a weaker but persistent expression in DLAV and ISVs that is enhanced by BNF treatment (C). This remaining vascular expression is lost in ahr1b,2 -/- and triple AHR mutants (D, E). Note the AHR-independent expression of cyp1a1 in the gut (arrows in E) Similar results are seen in the BNF-induced cyp1b1 expression, which is weakly maintained in ISVs and DLAV of ahr2 mutants and lost in ahr1b,2 -/- and triple AHR mutant embryos (F-J). Numbers of embryos analyzed are the same as in Fig 4 (cyp1a1) and Fig 5 (cyp1b1). All scale bars are 100 um. Abbreviations–AHR: aryl hydrocarbon receptor, BNF: beta-naphthoflavone, CYP: cytochrome p450, DA: dorsal aorta, DLAV: dorsal longitudinal anastomotic vessel, DMSO: dimethylsulfoxide, hpf: hours post fertilization, ISV: intersegmental vessel, PCV: posterior cardinal vein, WT: wildtype. (TIF) [file pone.0183433.s003.tif]
